# Structure, physicochemical properties, and hypolipidemic activity of soluble dietary fiber obtained from button mushroom (*Agaricus bisporus*)

**DOI:** 10.1016/j.fochx.2025.102657

**Published:** 2025-06-12

**Authors:** Xueqi Zhao, Yajie Zhang, Xuerui Wang, Liang Yao, Yuling Qu, Fengyun Zhao, Jianmin Yun

**Affiliations:** a College of Food Science and Engineering, Gansu Agricultural University, Lanzhou 730070, Gansu, China; b Gannong Moli (Qingyang) Agricultural Development Co., Ltd, Qingyang 745000, Gansu, China

**Keywords:** *Agaricus bisporus*, soluble dietary fiber, structural characterization, hypolipidemic activity, HepG2 cell model validation

## Abstract

The purpose of this paper was to investigate the structural characteristics, physicochemical properties, and hypolipidemic activity of soluble dietary fiber from button mushroom (BMSDF). Our results show that BMSDF is mainly composed of galactose, glucose and mannose, has the typical spectral characteristics of dietary fiber (FT-IR), characteristic diffraction curves (XRD), and the typical characteristics of polysaccharide nuclear magnetic hydrogen spectrum, which contained both α and β glycosidic bonds. The loose and porous microstructure of BMSDF observed under scanning electron microscopy makes it has good water holding capacity, oil holding capacity, cholesterol adsorption capacity, bile salt binding capacity. The lipid metabolism-related enzymes inhibitory assay results showed that 1.2 mg/mL BMSDF reached 75.29 % of the effect of orlistat and the inhibition of cholesterol esterase reached 37.05 %. And 100 μg/mL BMSDF reduces lipid deposition, significantly decreases the content of triglyceride and serum total cholesterol in oleic acid-induced HepG2 high-fat cells, while improves abnormal liver function enzyme activity, exhibiting good hypolipidemic biological activity.

## Highlights

• The loose porous morphology makes it have better oil holding characteristics.
• BMSDF can adsorb cholesterol in food and increase the ability to bind bile salts.
• BMSDF exerts a better hypolipidemic activity in lipid adsorption test.
• BMSDF can inhibit the activity of lipid metabolism-related enzymes.
• Verify the hypolipidemic activity of BMSDF by the high-fat HepG2 cell model.

## Graphical abstract

**Figure:**
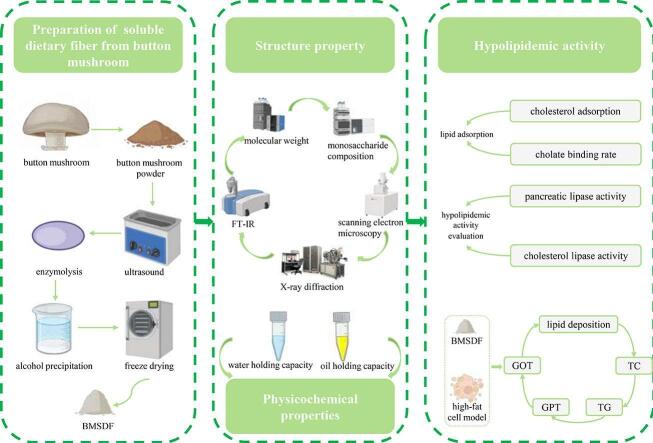

## 1 Introduction

Dietary fiber is a complex polysaccharide, which cannot be digested and absorbed by the human body due to the lack of specific enzymes (Yan et al., 2024). Dietary fiber has been recognized as the seventh important nutrient in addition to carbohydrates, proteins, fats, water, vitamins, and minerals (Dahl & Stewart, 2015). According to its solubility in water, dietary fiber can be divided into soluble dietary fiber (SDF) and insoluble dietary fiber (IDF) (Xiong et al., 2023). IDF mainly includes cellulose, hemicellulose, lignin, chitin, and similar components (Wu et al., 2020). SDF mainly includes oligosaccharides, pectin, β-glucan, and galactomannan (Zhu et al., 2019). Among them, SDF has been the subject of extensive research due to its significant physiological properties. Studies have found that dietary fiber can lower the occurrence rate of obesity, cardiovascular disease, cancer, and type II diabetes (Cassidy et al., 2018). At present, atherosclerosis, coronary heart disease, hyperlipidemia, and other diseases have become the main diseases that endanger human health as living standards rise (Zhang, Wu, et al., 2023). Among them, hyperlipidemia represents a significant risk factor for atherosclerosis and coronary heart disease.

Numerous studies have indicated significant differences in the structural characteristics and functional activities of dietary fibers obtained from different sources and preparation methods. Previous studies on dietary fiber have mainly focused on plant raw materials, such as orange peel (Wang et al., 2015), bamboo shoots (Tang et al., 2022), and sugarcane (Gil-López et al., 2019). For example, Wang Kunli et al. extracted two kinds of dietary fiber from kiwifruit via enzymatic, acid, and alkali extraction methods. Using comparative analysis, it was found that the monosaccharide composition of dietary fiber extracted by these three methods was basically the same, but there were differences in yield, purity, physical and chemical properties, and hypoglycemic activity (Wang, Yin, et al., 2021). However, research on dietary fiber derived from macrofungi has gradually attracted attention in recent years.

Edible fungi now hold a more and more crucial place in people's dietary recipes as homologous food and medicine ingredients. As one of the main components of edible fungi, dietary fiber can potentially be developed into a natural hypolipidemic active component. There is evidence that dietary fiber from *Flammulina velutipes* has a significant improvement effect on the weight, blood lipid, and serum antioxidant of high-fat obese model mice (Wang et al., 2022). Dong Yuehua et al. studied the effects of SDF obtained from three edible fungi (*Hericium erinaceus*, *Lentinula edo*des, and button mushroom) extracted using water extraction and alcohol precipitation on human intestinal flora. They found that these three dietary fibers can affect the richness of intestinal microorganisms to varying degrees and there were some differences in improving the intestinal environment (Dong et al., 2024). Yu et al. used single factor experiment and response surface method to analyze the structure, physicochemical and functional properties of soluble dietary fiber from *H. erinaceus*, and determined the optimal extraction process of soluble dietary fiber from *H. erinaceus* by ultrasonic-assisted enzymatic extraction (Yu et al., 2024). Flaga et al. investigated the method of obtaining soluble dietary fiber from the mycelium of *A. bisporus* using an alkaline oxidation treatment process (Fraga & Nunes, 2020). However, limited research exists on the structural characterization, physical and chemical properties, and biological activity evaluation of dietary fiber obtained from the fruiting body of button mushroom.

Button mushroom is the most widely cultivated edible fungus in the world. It is rich in dietary fiber, protein, and vitamins, and has a low fat content. Button mushroom has high economic value with edible and medicinal value (Usman et al., 2021). In this study, the fruiting body of *Agaricus bisporus* A15 was used as experimental material and the structure and physicochemical properties of button mushroom soluble dietary fiber (BMSDF) extracted using an ultrasonic-assisted complex enzymatic method analyzed. The in vitro hypolipidemic activity was evaluated based on the oleic acid-induced steatosis model of HepG2 cells and the role and mechanism of BMSDF in regulating lipid metabolism discussed. This study provides a theoretical basis and support for the further development of button mushroom dietary fiber products.

## 2 Materials and methods

### 2.1 Materials and reagents

Button mushrooms were provided by Gannong Moli Agricultural Development Co., Ltd. (Qingyang, Gansu) and was stored at 4 °C for subsequent use.

Trypsin (4000 U/mg) and α-amylase (4000 U/g) were purchased from Shanghai Yuanye Biotechnology Co., Ltd. Human liver cancer cells (HepG2) were purchased from Suzhou Starfish Biotechnology Co., Ltd. All chemicals and reagents used in this test were of analytical grade.

### 2.2 Preparation of soluble dietary fiber from button mushroom

**Fig. 1:**
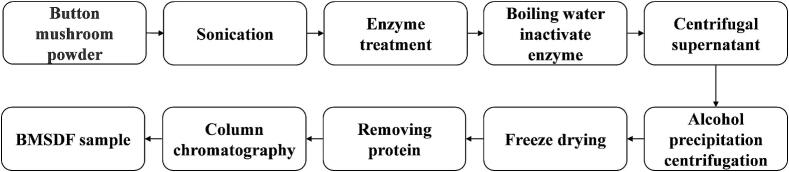
Flow chart for the preparation of soluble dietary fiber from button mushroom.

BMSDF was prepared by ultrasound-assisted composite enzymatic method as shown in Fig. 1. 20 g button mushrooms powder was mixed with 300 mL of distilled water at a material to liquid ratio (g∶mL) of 1∶15. After ultrasonic treatment at 30 °C for 50 min with 300 W power. Subsequently, 0.1 % trypsin was introduced and incubated in a water bath at 60 °C for 30 min. Afterwards, 5 % thermostable α-amylase was introduced and incubated at 95 °C for 35 min. The enzyme was subsequently inactivated by placing it in a boiling water bath for 10 min, followed by cooling to 60 °C. After that 0.5 mol/L NaOH aqueous solution was used to adjust the pH to 10 followed by a 1 h extraction. The pH was modified to 6.5 using 1 mol/L hydrochloric acid. Following centrifugation, the supernatant was combined with an aqueous solution of 95 % ethanol at four times the volume. After 4 h of alcohol precipitation, the sample was centrifuged at 4000 r/min for 10 min to collect the precipitate. And then pre-freezing at −80 °C for 24 h, vacuum freeze-drying was performed. The temperature of cold hydrazine was −50 °C, the vacuum level was 60 Pa, and freeze-drying took place for 24 h. Finaly the protein in sample was removed by Savage method, and BMSDF sample was obtained after purifing by DEAE-52 chromatography column and Sephadex G-50 gel column.

### 2.3 Structural composition of soluble dietary fiber from button mushroom

#### 2.3.1 Molecular weight

BMSDF was dissolved in 0.1 mol/L NaNO_3_ (the mass fraction of NaNO_3_ was 0.02 %, *w*/w) to make the final concentration of the sample reach 1 mg/mL. The molecular weight of BMSDF was detected using a gel chromatography-differential multi-angle laser light scattering system according to the method of Qin (Qi et al., 2023). It is filtered through a membrane with a pore size of 0.45 μm. The column temperature was 45 °C, the injection volume was 100 μL, the flow rate was 0.6 mL/min, and the isocratic elution was 75 min.

#### 2.3.2 Monosaccharide composition

The monosaccharide composition of BMSDF was analyzed using high performance anion exchange chromatography (Li et al., 2019). The BMSDF sample was mixed with 1 mL 2 mol/L trifluoroacetic acid and heat it at 121 °C for 2 h. After nitrogen blow drying, methanol was added for washing operation, and then dried. This step was repeated 2 to 3 times. Dissolved with sterile water and filtered with 0.22 μm microporous filter membrane for testing. The column temperature was 30 °C, the injection volume was 5 μL, the flow rate was 0.5 mL/min, and gradient elution was performed.

#### 2.3.3 Scanning electron microscopy (SEM)

The BMSDF sample of button mushroom was fixed on the copper pile with conductive adhesive to form a thin layer. The excess dietary fiber powder was removed by ear washing ball, and the gold was sprayed by ion sputtering. S-3400 N scanning electron microscope (Hitachi, Japan) was used, and the acceleration voltage was 15 kV. Then the two magnifications of 1000 times and 2000 times were selected to observe and photograph the SDF of button mushroom (Zhong et al., 2021).

#### 2.3.4 X-ray diffraction (XRD)

The crystal structure of BMSDF samples was analyzed by XRD. The dried BMSDF were fully crushed and analyzed by XRD after passing through a 100-mesh sieve. Operating conditions: copper target, tube voltage 40 kV, current 40 mA, step size 0.04°. The scanning speed was controlled at 17.7 s/step, the scanning interval is 2θ between 5° and 60° (Zhang et al., 2017).

#### 2.3.5 Fourier transform infrared (FT-IR) spectroscopy

The samples were analyzed by infrared spectrometer, and the functional groups of BMSDF were identified and the configuration of glycosidic bond was preliminarily judged. Precisely 1.5 mg of the BMSDF sample was weighed. 100 mg of dried potassium bromide powder was incorporated with the sample and the resulting mixture thoroughly ground until no crystals were visible. After being pressed into transparent sheets by a tableting machine, FT-IR was used to scan and determine in the range of 400–4000 cm^−1^ (Berktas & Cam, 2025).

#### 2.3.6 Nuclear magnetic resonance (NMR) spectroscopy

20 mg BMSDF sample was dissolved in 1.5 mL D_2_O and oscillated to achieve complete dissolution. It was placed in a nuclear magnetic tube and kept at room temperature for 12 h. Then, one-dimensional NMR spectra (^1^H and ^13^C) were measured using a 600 MHz NMR instrument (ASCEND™600, Brooke, China), and the chemical shifts were expressed in ppm (Dai et al., 2019).

### 2.4 Physicochemical properties of soluble dietary fiber from button mushroom

#### 2.4.1 Water holding capacity (WHC)

Refer to Zhang Jie's method and modify it (Zhang, Xu, et al., 2023), The BMSDF was placed in a centrifuge tube and shaken with distilled water for 1 h, then to calculate the WHC:

(1) $$\mathrm{WHC}\;\left(g/g\right)=\frac{m1-m0}{m}$$

m_1_: Total weight of sample and centrifuge tube after water absorption, g.

m_0_: Centrifuge tube weight, g.

m: Sample mass, g.

#### 2.4.2 Oil holding capacity (OHC)

Following Yan's method (Yan et al., 2018). put 0.5 g of button mushroom SDF in a centrifuge tube. Peanut oil was added to it and centrifuged after reaction. Then to calculate OHC:

(2) $$\begin{array}{c} \mathrm{OHC}\;\left(g/g\right)=\frac{m1-m-m0}{m} \end{array}$$

m_1_: Total weight of sample and centrifuge tube after water absorption, g.

m_0_: Centrifuge tube weight, g.

m: Sample mass, g.

### 2.5 In vitro hypolipidemic activity of soluble dietary fiber from button mushroom

#### 2.5.1 Cholesterol adsorption (CA)

The CA was determined according to the method of Zheng (Zheng et al., 2018) with some modification. The standard curve of the cholesterol mass concentration was obtained using a cholesterol standard solution and o-phthalaldehyde. Add distilled water to the egg yolk and stir until turbid. A total of 0.5 g BMSDF sample was added to 15 mL emulsion and stirred evenly, and the pH was adjusted to 2.0 and 7.0 for gastrointestinal simulation. After shaking at 37 °C for 2 h, 0.15 mL of the supernatant was added to 0.35 mL of glacial acetic acid, 1.5 mL of o-phthalaldehyde solution and 1 mL of concentrated sulfuric acid. After mixing and shaking, the supernatant was placed at room temperature for 10 min, and the absorbance was measured at 550 nm:

(3) $$\begin{array}{c} \mathrm{CA}\;\left(\mathrm{mg}/g\right)=\frac{m1-m2}{m0} \end{array}$$

m_1_: Cholesterol quality in emulsion (mg), mg.

m_2_: Cholesterol quality in supernatant, mg.

m_0_: Sample mass, g.

#### 2.5.2 Cholate binding rate (CBR)

The CBR was refer to the Huang's method (Huang et al., 2021). The mass concentration standard curve of sodium cholate was obtained using a sodium cholate standard solution and furfural. 0.2, 0.4, 0.6, 0.8, and 1.0 g of BMSDF powder were taken in the test tube, and add distilled water to 10 mL. Then 4 mL of 2 mg/mL sodium cholate was added to each sample and shaken in a thermostatic oscillator at 37 °C for 2 h. The shaken mixture was transferred to a centrifuge tube and centrifuged at 4000 r/min for 20 min. The CBR was calculated after centrifugation as follows:

(4) $$\begin{array}{c} \mathrm{CBR}\;\left(\%\right)=\frac{1-c1}{c2}\times 100\% \end{array}$$

c1: Unconjugated bile salt concentration in the experimental group, mol/L;

c2: Total bile salt concentration in blank control group, mol/L.

#### 2.5.3 Pancreatic lipase inhibition ability

Refer to Gutiérrez's method (Gutiérrez-Grijalva et al., 2019). Preparation of 0.5 mg/mL pancreatic lipase solution, 0.5 mg/mL 4-methylumbelliferone oleic acid ester solution for later use; 40 mg BMSDF samples were weighed and prepared into 10 mg/mL solution, and different concentration gradients were set. With orlistat as the positive control of the experiment, and at an excitation wavelength of 320 nm and an emission wavelength of 450 nm (See Table 1). The calculation formula of inhibition rate is:

(5) $$\begin{array}{c} \text{Inhibitory rate}\;\left(\%\right)=1-\frac{A-B}{C-D}\times 100 \end{array}$$

**Table 1:** Pancreatic lipase activity inhibition reaction system.

Grouping	Enzyme (mL)	Specimen (mL)	4-Methylumbelliferone oleate (mL)	PBS (mL)
sampling group(A)	1	4	1	0.5
Sample control group(B)	PBS	4	1	0.5
blank group(C)	1	PBS	1	0.5
blank control group(D)	PBS	PBS	1	0.5

#### 2.5.4 Cholesterol esterase inhibition

Refer to Chen's method (Chen et al., 2025) and adjust it. A 4 mmol/L PNPB solution was prepared, and the pH of PB buffer (containing 5.16 mmol/L sodium taurocholate and 0.1 mol/L sodium chloride) was adjusted to 7 for later use. In a 15 mL centrifuge tube, 0.05 mL of 20 μg/mL cholesterol esterase solution and 0.05 mL of BMSDF (50,100,200,400,800,1000 μg/mL) and 1 mL of PB were added in turn to maintain 10 min at 25 °C, and 0.02 mL of PNPB solution was added to maintain 30 min at 25 °C. Repeat three times in each group. After the end of the experiment, 200 μL of the reaction liquid was sucked out from each tube and placed in a 96-well plate (See Table 2). The absorbance is measured at 405 nm:

(6) $$\begin{array}{c} \text{Inhibitory rate}\;\left(\%\right)=1-\frac{A-B}{C-D}\times 100 \end{array}$$

**Table 2:** Cholesterol esterase activity inhibition reaction system.

Grouping	Enzyme (mL)	Specimen (mL)	PNPB (mL)	PB (mL)
sampling group (A)	0.05	0.05	0.02	1
Sample control group (B)	PB	0.05	0.02	1
blank group (C)	0.05	PB	0.02	1
blank control group (D)	PB	PB	0.02	1

### 2.6 Hypolipidemic cell model

#### 2.6.1 Cytotoxicity test

HepG2 cells were observed microscopically. HepG2 cells with good growth and density greater than 80 % were selected, collected, and then cultured for 24 h (Krochmal-Marczak et al., 2021). After discarding the original medium, different concentrations of BMSDF (12.5, 25, 50, 100, 200, and 400 μg/mL) and different concentrations of OA (100, 200, 300, 400, and 500 μM) were added for 48 h. The cell viability was assessed using a CCK-8 kit. (Biosharp, Beijing, China). HepG2 without BMSDF and OA was used as a blank control.

#### 2.6.2 Establishment of high-fat cell model

Different concentrations of OA in 2 % FBS DMEM medium were used to induce the culture for 24 h and 48 h, respectively. Lipid deposition in the cells was observed after oil red O staining. The relative content of lipids in HepG2 cells was calculated and the optimal OA concentration was determined.

#### 2.6.3 Lipid deposition

Refer to the method of Sun et al. for cell grouping (Sun et al., 2019): The experiment was divided into the control group: cells + DMEM medium with 2 % FBS; model group: cells +400 μM OA + 2 % FBS DMEM medium; each sample group (25, 50, and 100 μg/mL) + 400 μM OA + 2 % FBS DMEM medium; positive drug group: simvastatin (50 μg/mL) + 400 μM OA + 2 % FBS DMEM medium.

Refer to the methods of Song Jia and make appropriate modification (Song et al., 2022). After 24 h of culture, the model group, each sample group, and the positive drug group were added with 400 μM OA for 48 h to construct a high-fat cell model and then DMEM, SDF, and simvastatin in 2 % FBS were added according to the cell grouping for 24 h. Discard the original medium. After fixed with paraformaldehyde, rinsed with PBS and isopropanol. Then dyeing with pre-prepared oil red O dye for 30 min.,60 % isopropanol prepared in advance was added to infiltrate once, and PBS buffer was used to repeatedly soak for 3 times. The cells were observed under an inverted microscope and photographed, and the lipid accumulation of the cells after staining recorded. After staining, the PBS buffer was discarded, and isopropanol was introduced to swiftly permeate the cells.

(7) $$\begin{array}{c} \text{Lipid change rate}\;\left(\%\right)=\frac{\mathrm{SOD}-\mathrm{NOD}}{\mathrm{NOD}}\times 100 \end{array}$$

#### 2.6.4 Triglycerides and serum total cholesterol

The cells were collected from each well after the being grouped and cultured according to section 2.6.3. After the cells were resuspended in 300 μL of PBS, 200 μL of the extract was added, and then cell crushing performed. A total cholesterol (TC) kit and triglyceride (TG) kit (Nanjing Jiancheng Co., Ltd., Nanjing, China) were employed to measure the TC and TG contents in the supernatant of the samples, respectively (Song et al., 2025). Measure protein content in cells using BCA assay kit.

#### 2.6.5 Liver function enzyme activity

After the cells were broken as described in section 2.6.4, the glutamic pyruvic transaminase (GPT) and glutamic oxalacetic transaminase (GOT) activity was determined using a GPT and GOT kit (Shanghai Preferred Biotechnology Co., Ltd., Shanghai, China). Measure protein content in cells using BCA assay kit.

### 2.7 Statistical analysis

At least 3 parallel control groups were set up in each experiment and the results presented as the mean ± SD. IBM SPSS Statistics 27 software was used to analyze the variance with Duncan multiple comparison for in-depth analysis. A *P* < 0.05 indicated a statistically significant difference. Origin 2021 by OriginLab was used for drawing.

## 3 Results and discussion

### 3.1 Structural composition of BMSDF

#### 3.1.1 Molecular weight

The molecular weight can affect the hydration characteristics, cation exchange capacity, texture, and application characteristics of dietary fiber (Li, Wang, et al., 2022). The relative molecular weight of BMSDF was 794.691 kDa, which was calculated using gel permeation chromatography of the BMSDF sample. The molecular weight of BMSDF is lower when compared with other dietary fibers. Lower molecular weight can improve the water holding capacity of dietary fiber, which is due to the low molecular weight makes more free hydroxyl exposed (Li, Hu, et al., 2022). This may be attributed to the fact that ultrasonic and alkali treatments cause the glycosidic bond of the macromolecular substances in BMSDF to break, which releases low-molecular-weight soluble polysaccharides, resulting in a decrease in the molecular weight (Ma et al., 2025).

#### 3.1.2 Monosaccharide composition

**Fig. 2:**
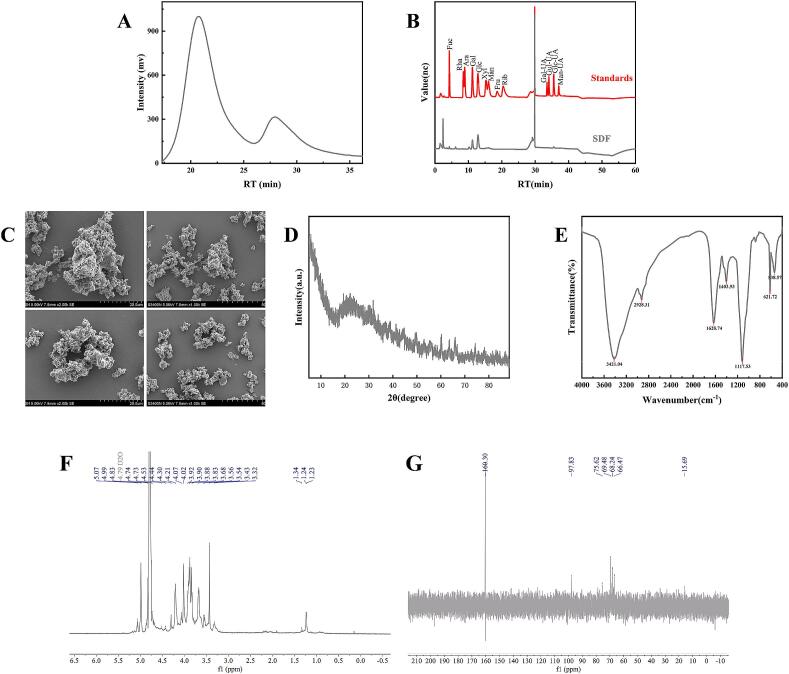
Analysis of the structural characteristics of soluble dietary fiber from button mushroom. (A) Molecular weight (detected by a gel chromatography-differential multi-angle laser light scattering system); (B) monosaccharide composition (analyzed by high performance anion exchange chromatography); (C) scanning electron microscopy; (D) Crystal structure identification (X-ray diffraction); (E) Analysis of functional groups and glycosidic bonds (Fourier transform infrared spectroscopy); (F) Nuclear magnetic resonance ^1^H spectrum; (G) Nuclear magnetic resonance ^13^C spectrum.

Fig. 2 shows the that BMSDF is composed of galactose (Gal), glucose (Glu), mannose (Man), galacturonic acid (Gal-UA)，fucose (Fuc), and xylose (Xyl). The molar ratio of the main monosaccharides was galactose:glucose:mannose = 26.33:53.32:6.09. Galacturonic acid is a polymer linearly linked by α-1,4 glycosidic bonds and is the main monosaccharide in pectin. Xylose is the main component of hemicellulose and exists in two dietary fibers (Qiu et al., 2017). This shows that BMSDF is a heteropolysaccharide composed of different sugar residues, which may contain pectin and hemicellulose. BMSDF has a similar monosaccharide composition to soluble dietary fiber obtained from pear pomace (Yu et al., 2024), but the molar ratio between different monosaccharides is different.

#### 3.1.3 SEM analysis

The microstructure of dietary fiber is related to its pore characteristics and effective surface properties (Ulbrich & Flöter, 2014). By observing the morphology of BMSDF utilizing SEM, it was found that the BMSDF sample shows an irregular block distribution, loose and porous, uneven surface morphology and high specific surface area (Fig. 2C). This loose and porous structure is due to the destruction of the structure by ultrasonic treatment and alkali treatment during the extraction process (Tang et al., 2024). Therefore, more hydrophilic groups and lipophilic groups are exposed on the surface of BMSDF, which are of great significance to its hydration and adsorption properties (Elleuch et al., 2011).

#### 3.1.4 XRD analysis

XRD analysis was used to assess alterations in the cellulose crystalline structure of the dietary fiber (Xia et al., 2025). Fig. 2D shows that BMSDF exhibits an obvious diffraction peak at 26.47°, which belongs to the characteristic X-ray diffraction curve of type I cellulose (Zhang et al., 2020). However, it exhibits irregular peaks at 26.47–50°, which may be due to the destruction of the strength and size of the crystal peak by the strong alkali solution (Hua et al., 2019). The XRD results of citrus fiber and bamboo shoot dietary fiber reported in other studies are different from the XRD patterns obtained for the BMSDF of the three fibers used in this study. In addition to the difference in the source of the fiber, it may be that the stronger physical and chemical treatment methods used in other studies modified the cellulose resulting in the diffraction peaks being more complex (Jurevičiūtė et al., 2022).

#### 3.1.5 FT-IR spectroscopy

The organic functional groups of SDF were identified by the Fourier Transform Infrared method (Chen et al., 2018). BMSDF exhibits similar spectral characteristics to other dietary fibers. The characteristic absorption peaks appear at 3370–3390 cm^−1^,1620–1633 cm^−1^, and 1415–1422 cm^−1^, but the absorption intensities were different, indicating that the BMSDF extracted from different materials still contain the functional groups of polysaccharides (Li, Wang, et al., 2017). The absorption peak at 3421 cm^−1^ was caused by the O—H stretching vibrations of cellulose and hemicellulose (Yin et al., 2022). The absorption peak at 2928 cm^−1^ originates from the C—H stretching vibrations of the polysaccharide methyl, methylene, and methylene groups, which are related to the content of hydrophobic groups in SDF (Lyu et al., 2021). The strong peak observed near 1628 cm^−1^ and the stretching vibrations of C=O in the carbonyl group indicate the presence of galacturonic acid. The weak peak observed near 1403 cm^−1^ corresponds to the vibration of COO^−^. The absorption peak observed at 1115 cm^−1^ corresponds to C—O, C—C, and C-O-C stretching in hemicellulose (Kang et al., 2024).

#### 3.1.6 NMR spectroscopy

The ^1^H NMR spectrum (Fig. 2F) shows that the signal of BMSDF is mainly concentrated in the range of *δ* 30–5.5 ppm, with typical characteristics of the proton NMR spectrum of polysaccharides (Hatzakis, 2019). In general, the signals of the anomeric hydrogens of- and β-glycosidic bonds are mainly distributed in the regions of *δ* 4.8–5.5 and *δ* 4.4–49 ppm, respectively (Zhou & Huang, 2023). Based on the ^1^H spectrum characteristics, combined with the analysis of its monosaccharide composition, the peak in the range of δ4.4–4.6 ppm was speculated to be the H1 of β-D-Gal or β-D-Glu; the signal at *δ* 4.7–4.9 ppm is speculated to be the anomeric proton of β-D-Man. The peak at 4.53 ppm indicated the presence of β-D-Xyl (Wang, Li, et al., 2021). The signal at 4.99 ppm and 5.07 ppm is presumed to be α-D-Gal-UA (Hu et al., 2023); the double peak at δ1.2–1.3 ppm is the characteristic peak of fucose C6 methyl. Therefore, it is proved that BMSDF contains both α and β glycosidic bonds (Huang et al., 2024). According to the literature of Jiang et al., the peaks between *δ* 63.36–76.70 ppm were attributed to C2, C3, C4, C5 on the unsubstituted sugar ring, and the signals at *δ* 90–110 ppm were assigned the substituted C1 (Jiang et al., 2019). Based on the previous reports (Cheng & Neiss, 2012; Garrido et al., 2021; Li, Li, et al., 2017), we inferred that the peaks in the range of *δ* 70–78 ppm in the ^13^C NMR spectrum of BMSDF (Fig. 2G) were glucose C2-C5, the peaks near *δ* 68–70 ppm were galactose substituted C4 or fucose C5, the peaks in the range of *δ* 65–67 ppm were speculated to be xylose5 methylene carbon, the peaks near *δ* 94–98 ppm were mannose isomeric head C1, and the characteristic low field signal of fucose C6 methyl carbon in the range of *δ* 15–18 ppm, and the peak at *δ* = 160.3 may be attributable the carboxyl carbon signal of galacturonic acid.

### 3.2 Physicochemical properties of button mushroom dietary fiber

**Fig. 3:**
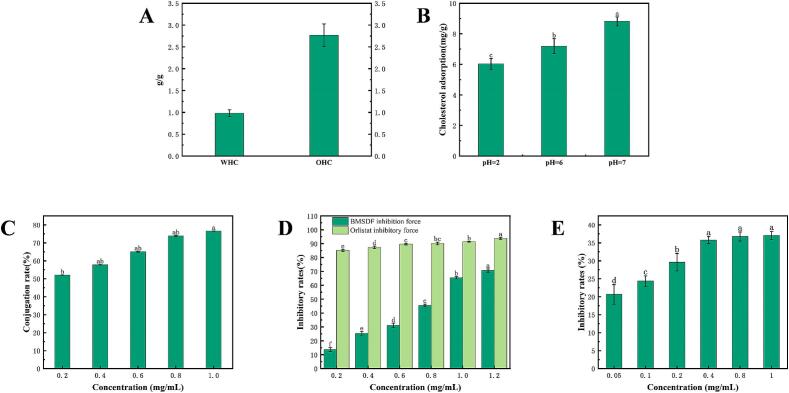
Analysis of the physicochemical properties and hypolipidemic activity of water-soluble dietary fiber from button mushroom. (A) Water holding capacity and oil holding capacity; (B) cholesterol adsorption capacity; (C) cholate binding ability; (D) pancreatic lipase inhibition ability; (E) cholesterol lipase inhibition ability.

Previous studies have shown that soluble dietary fiber can absorb greater amounts of lipids from both food and the digestive system, which effectively decreases the risk of hyperlipidemia (Ji et al., 2024). Our morphological observations using SEM show that the structure of BMSDF was loose and the porosity was high (Fig. 3A). The porous structure exposes more hydrophilic and lipophilic groups, facilitating the diffusion of oil and water molecules into BMSDF, so that it has a better WHC and OHC (Li et al., 2024). The oil binding capacity is one of the main parameters used to assess the ability of SDF to accelerate lipid excretion and lower serum cholesterol levels (Elleuch et al., 2011).

### 3.3 In vitro hypolipidemic activity of button mushroom soluble dietary fiber

#### 3.3.1 Cholesterol adsorption

High cholesterol can induce cardiovascular disease. Dietary fiber can prevent the absorption of cholesterol and the pH value has a significant effect on the adsorption of cholesterol by dietary fiber (Liu et al., 2016). Therefore, pH = 2 and 7 were used to simulate the cholesterol adsorption effect of dietary fiber in the different digestive tracts of the human body. Fig. 3B shows that the cholesterol adsorption effect at pH = 7 was significantly higher than that at pH = 2. This can be attributed to the higher concentration of H^+^ in an acidic environment, which results in mutual repulsion between the cholesterol molecules and BMSDF, reducing the adsorption of cholesterol by dietary fiber.

#### 3.3.2 Cholate binding

Some previous studies have shown that substances such as cellulose and pectin can be combined with bile salts to form complexes, thereby preventing the formation of small cholesterol particles and reducing the absorption of cholesterol (Wu et al., 2020). And dietary fiber can adsorb bile salts in the liver, making cholesterol faster (Zhang et al., 2011). Fig. 3C shows that with an increase in the BMSDF concentration, the binding rate of sodium cholate gradually increases. When the dietary fiber concentration reached 1.0 mg/mL, the binding rate of sodium cholate can reach 76.51 %, indicating that BMSDF exhibits a strong binding ability toward bile salts.

#### 3.3.3 Lipid metabolism enzyme inhibition ability

Pancreatic lipase is essential for triglyceride digestion and inhibits the intestinal uptake of dietary triglycerides (Xue et al., 2020). Inhibition of pancreatic lipase activity has a beneficial effect on reducing fat absorption, which is a crucial method used to manage hyperlipidemia and obesity. Moreover, the hydrolysis of cholesterol ester in the human body is further inhibited by inhibiting the activity of cholesterol esterase, which plays a role in hypolipidemic. Fig. 3D and F show the inhibition rate of BMSDF on pancreatic lipase increases with an increase in its concentration, showing a good linear relationship. At the maximum concentration, the inhibition rate was 70.63 %, which was close to the inhibition rate of orlistat on pancreatic lipase. At the same time, the inhibition of BMSDF on cholesterol lipase also exhibits a good linear relationship. At a BMSDF concentration of 0.4 mg/mL, the inhibition of cholesterol lipase reached a higher level. This inhibitory test proved that the soluble dietary fiber of button mushroom shows a good inhibitory effect toward the two lipid metabolism enzymes.

### 3.4 Verification of the cell model for the hypolipidemic activity of dietary fiber from button mushroom

#### 3.4.1 Cytotoxicity test

**Fig. 4:**
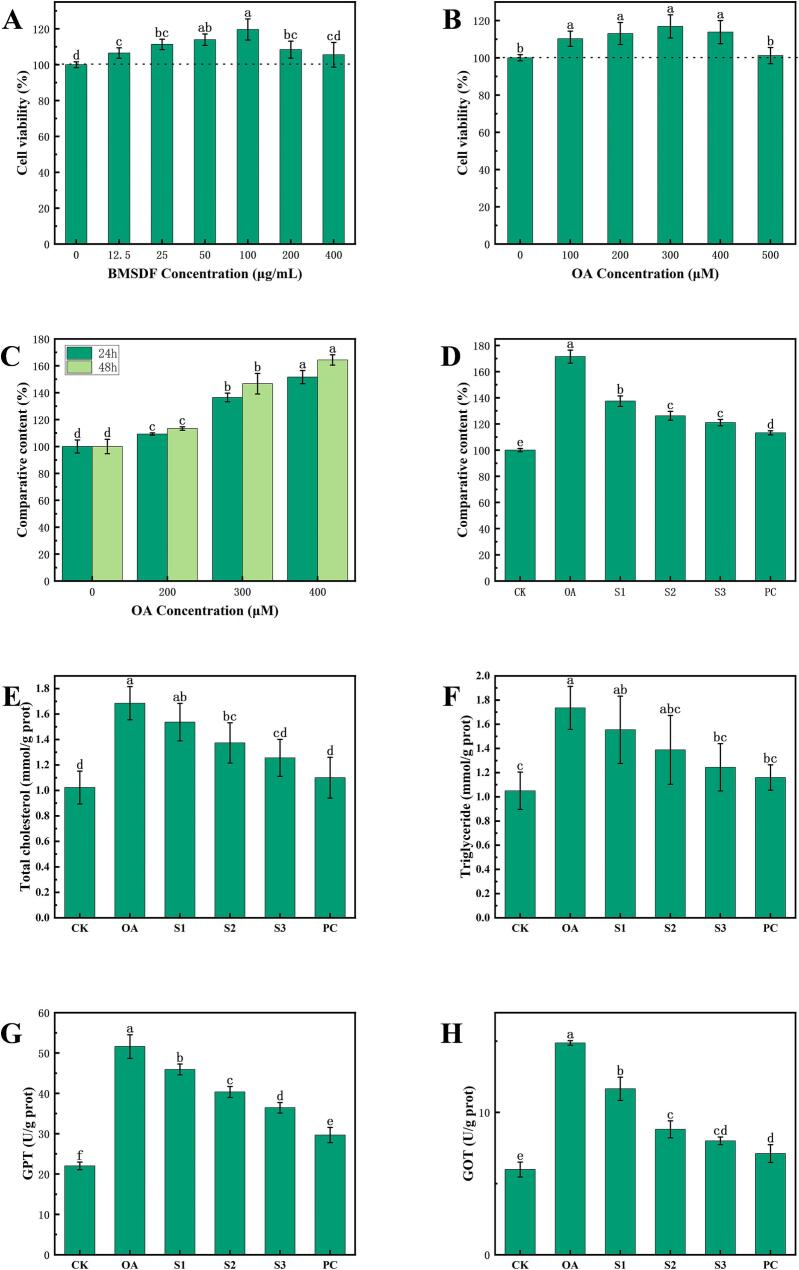
Analysis of the hypolipidemic activity of soluble dietary fiber from button mushroom in vitro. (A) The effect of SDF on the cell viability; (B) the effect of OA on the cell viability; (C) selection of the OA concentration in the construction of the high-fat cell model; (D) cell lipid deposition; (E) total cholesterol content; (F) triglyceride content; (G) determination of the alanine aminotransferase activity; (H) determination of the aspartate aminotransferase activity.

Fig. 4A shows BMSDF treatment below 100 μg/mL has a certain effect on the proliferation of HepG2 cells in contrast to the control group. When the concentration of BMSDF was greater than 100 μg/mL, the survival rate of HepG2 cells was inhibited. Therefore, BMSDF with concentrations of 25, 50, and 100 μg/mL were selected for our subsequent experiments.

Fig. 4B shows treatment with ≤400 μmol/L OA had no significant inhibitory effect on HepG2 cells when compared with the control group, while treatment with 500 μmol/L OA showed a significant inhibitory effect on HepG2 cells. Therefore, treatment with ≤400 μmol/L OA was selected for model screening to construct a high-fat cell model.

#### 3.4.2 Establishment of high-fat model

**Fig. 5:**
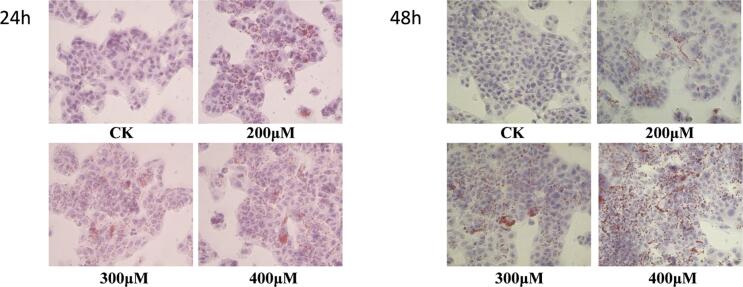
Effects of OA with different concentrations and different treatment times on lipid formation in HepG2 cells induced by OA.

According to the experimental results described in section 3.4.1, treatment with 200, 300, and 400 μmol/L OA was carried out for 24 h and 48 h, respectively. The accumulation of lipid droplets in the cells was observed under an inverted microscope after oil red O staining and the relative content of lipid droplets in the cells determined. Our results showed that the amount of lipid droplets in the cells gradually increased with an increase in the OA concentration (Fig. 5).

Fig. 4C show upon calculating the lipid content, the higher the OA concentration, the higher the lipid content. When combined with the experimental results described in section 3.4.1, it can be seen that after OA induction for 48 h, the lipid content of the cells reached 1.68 times that of the PC group, and the inhibitory effect on the survival rate of HepG2 cells was the smallest. Therefore, treatment with 400 μmol/L OA for 48 h was used to set up the induce steatosis model in HepG2 cells.

#### 3.4.3 Lipid deposition

**Fig. 6:**
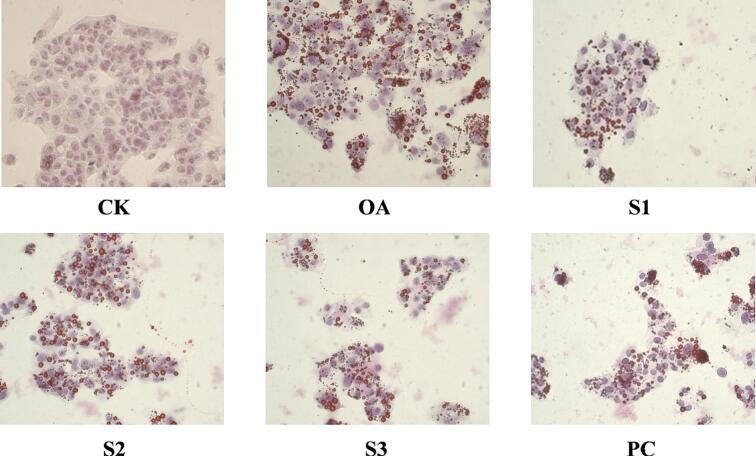
Effect of BMSDF treatment on lipid deposition in HepG2 cells.

Fig. 4D and Fig. 6 show that after being treated with 25, 50, and 100 μg/mL BMSDF, the red lipid droplets in HepG2 cells show a decrease in number, volume, and color. The relative content of intracellular lipids was also significantly improved in the model group. The hypolipidemic effect was more obvious with an increase in the BMSDF concentration and the hypolipidemic effect of 100 μg/mL BMSDF was the best.

#### 3.4.4 Triglyceride and serum total cholesterol

Fig. 4E and Fig. 4F show the contents of cholesterol and triglyceride in the HepG2 high-fat cell model induced by OA were higher than that in the control group. After treatment with different concentrations of BMSDF, the total cholesterol and triglyceride levels of each treatment group were significantly decreased, and the effect of 100 μg/mL BMSDF group was the most significant. This indicates that BMSDF treatment can effectively alleviate the high-fat state of HepG2 cells after OA induction.

#### 3.4.5 Liver function enzyme activity

The liver is the central organ of lipid metabolism. The activities of GPT and GOT are important indicators for evaluating liver function (Wu et al., 2018). The GPT and GOT activity in cells can reflect the degree of liver cell damage. Our results show that the content of GPT and GOT in the OA treatment group was significantly different from that in the CK group (in Fig. 4G and H). After being treated with OA, HepG2 transaminase begins to be released. After being treated with BMSDF, the levels of these two liver function enzymes are significantly decreased. The effect of the S3 group was the most obvious, approaching the positive PC drug group. This shows that BMSDF treatment can improve the liver function damage of HepG2 cells.

## 4 Conclusions

The structural characteristics and hypolipidemic activity of BMSDF extracted using an ultrasound-assisted compound enzyme method have been explored. Our results show that BMSDF has a lower relative molecular mass and was mainly composed of six monosaccharides, including galactose, glucose, and mannose with different molar ratios. BMSDF exhibits a porous structure under SEM, which gives it better water holding and oil holding characteristics, thereby reducing the absorption of cholesterol by the human body. Meanwhile, the effects of BMSDF on the lipid deposition, TC and TG contents, and liver function enzyme indexes in HepG2 cells has been verified using an oleic acid-induced HepG2 cell steatosis model test. It was confirmed that BMSDF exerts its hypolipidemic effect and mechanism by adsorbing excess lipids in the body and regulating lipid metabolism-related enzyme activities. This finding not only shows that BMSDF has the potential to develop functional foods, but also provides a new solution for the clinical development of natural substance products with hypolipidemic function to replace traditional statins for the treatment of hyperlipidemia, and thus provide a theoretical basis for the development and application of BMSDF in regulating lipid metabolism functional foods and drugs.

## CRediT authorship contribution statement

**Xueqi Zhao:** Writing – original draft, Validation, Methodology, Data curation. **Yajie Zhang:** Software, Data curation. **Xuerui Wang:** Resources, Formal analysis. **Liang Yao:** Funding acquisition. **Yuling Qu:** Supervision. **Fengyun Zhao:** Visualization. **Jianmin Yun:** Writing – review & editing, Project administration, Conceptualization.

## Declaration of competing interest

The authors declare that they have no known competing financial interests or personal relationships that could have appeared to influence the work reported in this paper.

## Data Availability

Data will be made available on request.
